# Binding of sulphonylureas to plasma proteins – A K_ATP_ channel perspective

**DOI:** 10.1371/journal.pone.0197634

**Published:** 2018-05-17

**Authors:** Peter Proks, Holger Kramer, Elizabeth Haythorne, Frances M. Ashcroft

**Affiliations:** Oxford Centre for Gene Function, Department of Physiology, Anatomy and Genetics, University of Oxford, Oxford, United Kingdom; Universidad Miguel Hernandez de Elche, SPAIN

## Abstract

Sulphonylurea drugs stimulate insulin secretion from pancreatic β-cells primarily by inhibiting ATP sensitive potassium (K_ATP_) channels in the β-cell membrane. The effective sulphonylurea concentration at its site of action is significantly attenuated by binding to serum albumin, which makes it difficult to compare *in vitro* and *in vivo* data. We therefore measured the ability of gliclazide and glibenclamide to inhibit K_ATP_ channels and stimulate insulin secretion in the presence of serum albumin. We used this data, together with estimates of free drug concentrations from binding studies, to predict the extent of sulphonylurea inhibition of K_ATP_ channels at therapeutic concentrations *in vivo*. K_ATP_ currents from mouse pancreatic β-cells and *Xenopus* oocytes were measured using the patch-clamp technique. Gliclazide and glibenclamide binding to human plasma were determined in spiked plasma samples using an ultrafiltration-mass spectrometry approach. Bovine serum albumin (60g/l) produced a mild, non-significant reduction of gliclazide block of K_ATP_ currents in pancreatic β-cells and *Xenopus* oocytes. In contrast, glibenclamide inhibition of recombinant K_ATP_ channels was dramatically suppressed by albumin (predicted free drug concentration <0.1%). Insulin secretion was also reduced. Free concentrations of gliclazide and glibenclamide in the presence of human plasma measured in binding experiments were 15% and 0.05%, respectively. Our data suggest the free concentration of glibenclamide in plasma is too low to account for the drug’s therapeutic effect. In contrast, the free gliclazide concentration in plasma is high enough to close K_ATP_ channels and stimulate insulin secretion.

## Introduction

Sulphonylureas are oral hypoglycaemic agents that are widely used in the management of type 2 diabetes and certain forms of monogenic diabetes. They reduce blood glucose levels by stimulating insulin secretion from pancreatic β-cells. Their primary target is the sulphonylurea receptor (SUR1) subunit of the ATP-sensitive potassium (K_ATP_) channel in the β-cell plasma membrane [1,2]. The structure of the K_ATP_ channel complex (Kir6.2/SUR1) in association with the sulphonylurea glibenclamide has recently been solved at atomic resolution [3]. The sulphonylurea binding pocket lies with the transmembrane domains, towards the inner side of the membrane, and comprises TMs 7, 8, and 11 on one side of the pocket and TMs15 and 17 on the other. Because sulphonylureas are lipid soluble [1], they are effective whether applied from the inside or outside of the membrane [4]. Drug binding induces K_ATP_ channel closure, which leads to membrane depolarisation, activation of voltage-dependent Ca^2+^ channels, and an influx of Ca^2+^ influx that triggers insulin granule exocytosis [5]. Sulphonylureas also stimulate insulin secretion independently of membrane potential, by interacting with proteins within the exocytotic pathway [6,7].

It is well established that sulphonylureas bind tightly to plasma proteins [8–10] and, as a consequence, their effective (free) concentration in plasma is much less than their total concentration. In order to determine the extent of block of K_ATP_ channel activity produced by a sulphonylurea *in vivo* from the concentration-response relation measured *in vitro*, it is therefore essential to correct for sulphonylurea binding to plasma proteins. Understanding the extent of drug block is of interest in order to answer a number of questions. For example, why do patients with neonatal diabetes due to activating K_ATP_ channel mutations require far higher drug doses than patients with type 2 diabetes? [11]. Why do patients with the more severe K_ATP_ channel mutations rarely suffer from hypoglycaemia, despite taking these very large doses? And finally, given the very tight binding of glibenclamide to plasma proteins, are plasma free glibenclamide levels sufficient to quantitatively explain the enhanced insulin secretion as entirely due to K_ATP_ channel inhibition? To answer these questions, it is necessary to know the concentration-response relation for sulphonylurea inhibition of K_ATP_ channel activity in the absence and presence of plasma proteins.

The *total* concentration of a drug in a body fluid such as plasma is usually given as the steady-state concentration (Css), which is the drug concentration at the time a dynamic steady state has been achieved, and rates of drug administration and elimination are equal. It is determined in a time-dependent manner by both its rate of metabolism and the apparent distribution volume. Sulphonylureas are predominantly distributed in the extracellular space bound to plasma proteins and are characterized by a low apparent distribution volume. Some sulphonylureas such as glibenclamide have active metabolites that may prolong their duration of action [12]. In addition, the steady-state *free* concentration of a drug is also determined by the extent to which is bound by plasma proteins.

The most abundant plasma protein is albumin, which is responsible for transporting various fatty acids, hormones and drugs in the circulation [13]. We therefore measured the effect of albumin on K_ATP_ channel block by two commonly used sulphonylureas–glibenclamide and gliclazide–which have very different rates of unbinding from SUR1: on the time course of electrophysiological experiments, gliclazide is fast, whereas glibenclamide is slow [14,15]. We also measured the binding affinity of glibenclamide and gliclazide to human plasma. We then estimated the circulating free drug concentration in human patients and in a mouse model of neonatal diabetes and used our data to predict the extent of sulphonylurea inhibition of K_ATP_ channels at therapeutic concentrations *in vivo*.

## Methods

### Use of animals

Use of animals for this research was approved by Animal Welfare & Ethical Review Board of the University of Oxford and by the UK Animals (Scientific Procedures) Act 1986 UK Home Office license number PPL 30/3198.

### Electrophysiology—Isolated mouse pancreatic β-cells

Pancreatic islets were isolated from C57BL/6 mice and dissociated into single cells according to established protocols [16]. Mice were killed by cervical dislocation. Cells were cultured in RPMI medium supplemented with 10% fetal calf serum, 11mmol/l glucose, 100U/ml penicillin and 100μg/ml streptomycin at 37°C in a humidified atmosphere of 5% CO_2_ in air. Whole-cell K_ATP_ currents were recorded from single cells 1–3 days after isolation, using the standard whole-cell configuration. The pipette solution contained (mmol/l): 107 KCl, 1 CaCl_2_, 1 MgCl_2_, 10 EGTA, 10 HEPES, 0.3 ATP (pH 7.2 with KOH). The extracellular solution contained (mmol/l) 138 NaCl, 5.6 KCl, 1.2 MgCl_2_, 2.6 CaCl_2_, 10 HEPES (pH 7.4 with NaOH). Gliclazide and bovine serum albumin (BSA) were added as stated in the text. Currents were recorded in response to alternate ±20mV depolarisations from a holding potential of –70mV applied at frequency of 0.3Hz, at 20–22°C. Currents were low-pass filtered at 1kHz, digitised at 5kHz using an Axopatch 200B (Axon Instruments) and analysed using pClamp10 (Axon Instruments) and Origin 8.5 (OriginLab Corporation).

Pancreatic islets are composed mainly of β-cells with much small numbers of α-cells and δ-cells. Given that the K_ATP_ channel is composed of Kir6.2 and SUR1 in all three cell types, there should be no difference in glibenclamide block. Indeed, no difference is observed in glibenclamide block of native channels in β-cells or recombinant channels expressed in *Xenopus* oocytes or mammalian cells [1, 14, 17, 18]. Nevertheless, we selected cells with K_ATP_ currents >100pA (whole-cell recordings), >40pA (perforated patch, βV59M β-cells) or >20pA (perforated patch, wild-type cells) to reduce contamination with α- and δ-cells, which have smaller K_ATP_ currents [19, 20].

### Electrophysiology—*Xenopus* oocytes

The time course of glibenclamide block is very slow at low drug concentrations, and difficult to distinguish from current rundown [1]. This problem becomes even more severe when the free drug concentration is decreased by binding to albumin. It was therefore not possible to measure glibenclamide inhibition of whole-cell K_ATP_ currents in pancreatic β-cells. We therefore used a different approach: we measured the single channel open probability (*P*_*O*_) in cell-attached patches on intact *Xenopus* oocytes expressing recombinant β-cell K_ATP_ channels. Channel activity was increased using the metabolic inhibitor Na-azide (3mmol/l).

Oocytes were surgically removed from *Xenopus laevis* frogs (European Xenopus Resource Centre, Portsmouth) anesthetized with 5g/l ethyl-m-aminobenzoate methanesulfonate (MP Biomedicals, France); subsequently, euthanasia was performed by decapitation. Macroscopic K_ATP_ currents were recorded from cell-attached and excised patches on *Xenopus* oocytes, heterologously expressing human Kir6.2 (Genbank NMOL/L000525 with E23 and I377) and rat SUR1 (Genbank L40624). Site-directed mutagenesis, preparation of mRNA, and isolation and injection of oocytes with mRNA were as described previously [21]. Currents were recorded at -60mV and 20–22°C, filtered at 5kHz, digitised at 20kHz using an Axopatch 200B and analysed using pClamp10 (Axon Instruments) and Origin 8.5 (OriginLab Corporation). The pipette solution contained (mmol/l): 140 KCl, 1.2 MgCl_2_, 2.6 CaCl_2_, 10 HEPES (pH 7.4 with KOH). The bath solution in cell-attached patches (and intracellular in excised patches) contained (mmol/l): 107 KCl, 1 CaCl_2_, 2 MgCl_2,_ 10 EGTA, 10 HEPES (pH 7.2 *wi*th KOH). Oocytes were pre-incubated for 30-60min prior to experiment in bath solution supplemented with 3mmol/l Na-azide, BSA (60mg/ml, or 0.9mmol/l) and glibenclamide as stated in the text.

### Electrophysiology—Data analysis

The single-channel open probability (*P*_*O*_) was estimated as

$$P_{0}=\frac{I_{MEAN}}{Ni}$$ Eq 1

where *I*_*MEAN*_ is the mean K_ATP_ current in cell-attached configuration, *N* is the number of active channels in the patch and *i* is the single-channel current (*i* = 4pA at -60mV). Following cell-attached recordings, the patch was excised and the number of active channels (*N*) was estimated from ~1s data stretches obtained once the K_ATP_ current had reached its maximum, using noise analysis [22].

The relationship between the sulphonylurea (*S*) concentration and the macroscopic current (*I)* or the single-channel open probability (*P*_*O*_*)* was fit with

$$\frac{I(S)}{I}or\frac{P_{o}(S)}{P_{0}}=a+\frac{1-a}{1+(\frac{\left[X\right]}{{IC}_{50}}{)}^{h}}$$ Eq 2

where *I(S)* is the macroscopic current and *P*_*O*_*(S)* is the cell-attached *P*_*O*_ in the presence of the drug, *I* and *P*_*O*_ are the macroscopic current and the cell-attached single channel open probability, respectively, in drug-free solution, *IC*_*50*_ is the drug concentration at which the inhibition is half maximal, *h* is the Hill coefficient and *a* is the fraction of K_ATP_ current remaining at drug concentrations that saturates the high-affinity binding site on SUR1 (*a* was set to 0 for glibenclamide). To correct for any rundown of whole-cell currents, control solutions braced each test concentration, and the control current is taken as the mean value of that before and after the test concentration.

### Chemicals and biological reagents

A 100mmol/l stock solution of gliclazide or glibenclamide in DMSO was prepared daily and diluted as required. Bovine serum albumin (BSA; Fraction V) was purchased from Roche Diagnostics, Mannheim, Germany. Fatty acid free BSA (Sigma-Aldrich, St Luis, USA) was used for insulin secretion experiments. The BSA concentration (0.9mmol/l; 60g/l) was chosen to approximate that of albumin in plasma (35-55g/l). Human plasma was purchased from Valley Biomedical Inc., Winchester, USA.

### Insulin secretion

Pancreatic islets were isolated from C57BL/6 mice using established protocols [16] and cultured overnight in RPMI-1640 (Sigma, UK) supplemented with 10mmol/l glucose, 100U/ml penicillin, 10μg/ml streptomycin and 10% fetal calf serum, at 37°C in a humidified atmosphere of 5% CO_2_/95% air. Mice were killed by cervical dislocation Insulin secretion was measured from triplicate batches of 10 islets incubated in 0.5 ml of Krebs-Ringer buffer (KRB) containing (mmol/l): 140 NaCl, 3.6 KCl, 1.5 CaCl_2_, 0.5 MgSO_4_, 0.5 NaH_2_PO_4_, 2 NaHCO_3_, 10 HEPES, and 0.1% (wt/vol) BSA, pH 7.4. Islets were washed twice and preincubated in 2mmol/l glucose KRB for 1 hour at 37°C, with gentle shaking. The pre-incubation buffer was discarded and islets were stimulated for a further 1 (or 2) hours with the test conditions indicated. An aliquot of the supernatant was collected, in order to quantify secreted insulin, and total insulin was extracted into 0.5 ml of acidified ethanol (75% (vol/vol) ethanol, 1.5% (vol/vol) 1 mmol/l HCl and 0.1% (vol/vol) Triton X-100). Secreted and total insulin concentrations were measured by ELISA (Mercodia, Uppsala, Sweden).

### Binding studies

The free plasma concentrations of sulphonylureas were determined in spiked plasma samples using an ultrafiltration approach [23]. For gliclazide, the following total drug concentrations (ng/ml) were used: 0, 1, 5, 20, 50, 200, 500, 1000, 2000, 3000, 4000. For glibenclamide, the total drug concentrations used were (ng/nl): 0; 10000; 50000. Aliquots (500μl) of spiked plasma samples were centrifuged in 50 KDa molecular weight cut-off centrifugal concentrators until >100μl ultrafiltrate was collected. Drug-free plasma ultrafiltrate was produced from blank plasma (10ml) and the obtained blank ultrafiltrate was used to prepare calibration standards and quality control (QC) samples. Sample extraction, analysis by liquid chromatography-mass spectrometry (LC-MS) and data analysis were carried out as described previously for total drug concentrations [24].

The *K*_*d*_ for sulphonylurea binding to albumin in human plasma was calculated according to the equation [25]:

$$C_{T}=C_{F}+\frac{n*[A]}{1+\frac{K_{d}}{C_{F}}}$$ Eq 3

where *C*_*T*_ and *C*_*F*_ are the total and free concentrations of the sulphonylurea, respectively, [A] is the albumin concentration (0.9mmol/l) and *n* is the number of sulphonylurea binding sites on albumin (for simplicity and for comparison with previous binding studies on isolated albumin, *n* = 1).

### Statistical analysis

All values are given as mean ± SEM. Statistical significance in electrophysiological experiments was determined using Student’s t-test. Insulin secretion was analysed using a one-way ANOVA followed by Tuckey post-hoc test to compare individual groups.

## Results

### BSA causes a small reduction in gliclazide block of the K_ATP_ channel

We first examined the effect of bovine serum albumin (BSA, 0.9mmol/l) on gliclazide block of whole-cell K_ATP_ currents in mouse pancreatic β-cells. Following establishment of the whole-cell configuration, the amplitude of the whole-cell K_ATP_ current first increases due to washout of intracellular ATP, and then slowly runs down with time. Gliclazide was tested once the maximal current amplitude had been reached, while the current was in a pseudo-steady state.

**Fig 1:**
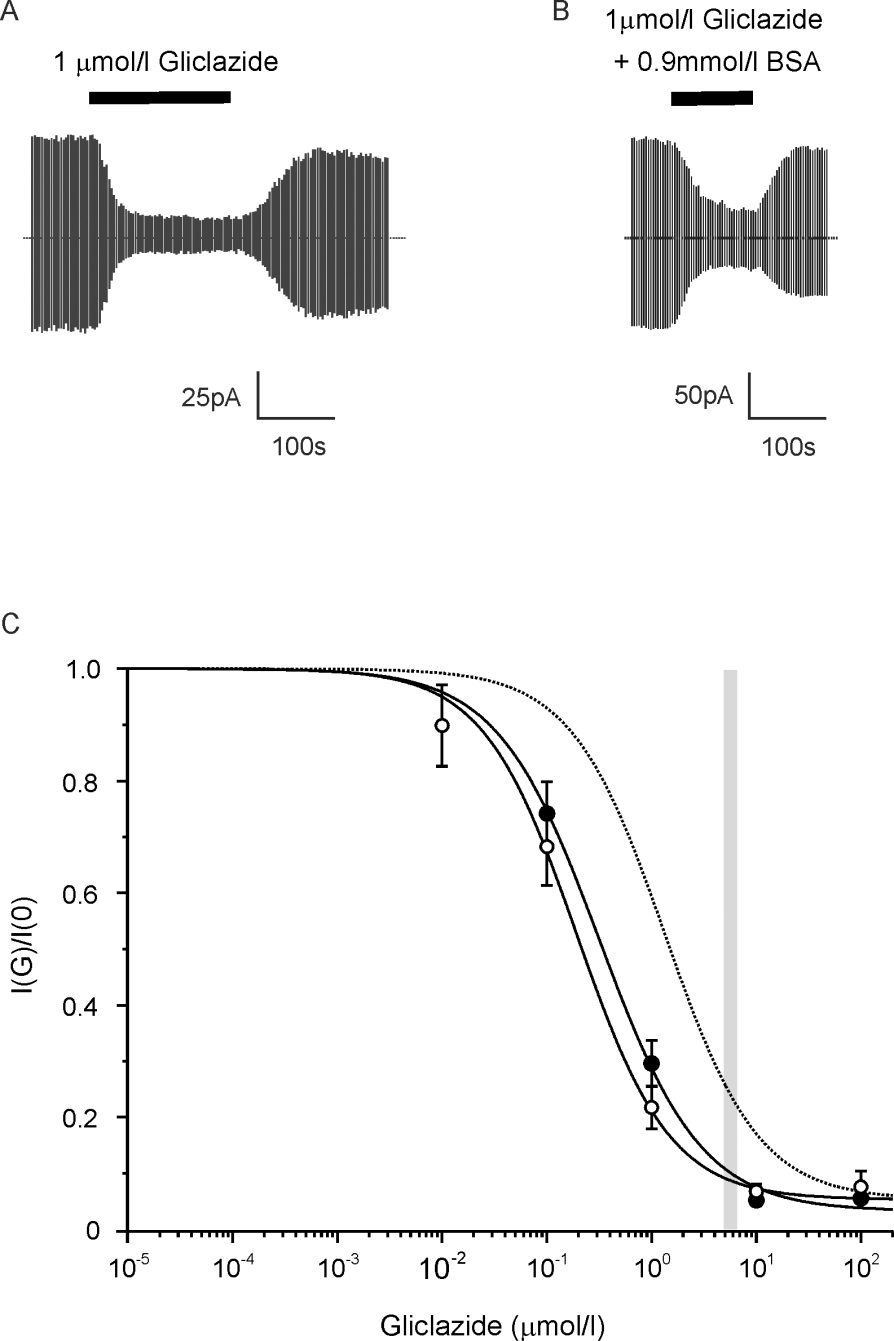
Effect of BSA on gliclazide block of the β-cell K_ATP_ current. (A,B) Representative whole-cell K_ATP_ currents recorded from mouse pancreatic β-cells in response to alternating ±20mV steps from a holding potential of -70mV. Gliclazide (1μmol/l) was added (as indicated by the bars) in the absence (A) or presence (B) of 0.9mmol/l BSA. The dotted line indicates the zero current level. (C) Concentration-response relationships for gliclazide inhibition of whole-cell K_ATP_ currents in mouse β-cells in the absence (○, n = 6) or presence (●, n = 5) of 0.9mmol/l BSA. Current is expressed relative to that in the absence of gliclazide. The solid lines are the best fit of Eq 2 to the mean data: (○) *IC*_*50*_ = 190nmol/l, *h* = 0.97, *a* = 0.05; (●) *IC*_*50*_ = 320nmol/l, *h* = 0.90, *a* = 0.03. This shift predicts 60% of drug is bound and a dissociation constant (*K*_*d*_) for drug binding to BSA of ~1.3mmol/l (Eq 3). The dotted line is the estimated gliclazide block in the presence of human plasma (HP) assuming gliclazide binds to plasma proteins with a *K*_*d*_ of 155μmol/l. The width of the grey bar indicates the mean C_SS_±SEM (steady-state total plasma concentrations of sulphonylurea drugs) of the total gliclazide concentration in the plasma estimated from a daily dose of 80mg and an AUC (the area under the plasma concentration against time curve) of 44μg.h/ml [26].

Fig 1A and 1B shows typical current traces recorded in response to 1μmol/l gliclazide in the presence and absence of 0.9mmol/l BSA, respectively. Fig 1C plots the corresponding concentration-inhibition relationships. BSA had a modest effect on gliclazide sensitivity, increasing the half maximal inhibitory concentration (*IC*_*50*_) from 190 to 320nmol/l. However, the effect did not reach significance.

### BSA dramatically reduces glibenclamide block of the K_ATP_ channel

We next investigated the effect of BSA on glibenclamide block of the K_ATP_ channel. For reasons outlined in the Methods, it was not possible to measure glibenclamide block of β-cell K_ATP_ currents in the same way as for gliclazide. Instead, we measured the single-channel open probability (*P*_*O*_) in cell-attached patches on intact *Xenopus* oocytes expressing recombinant β-cell K_ATP_ channels. Channel activity was increased using the metabolic inhibitor Na-azide (3mmol/l).

**Fig 2:**
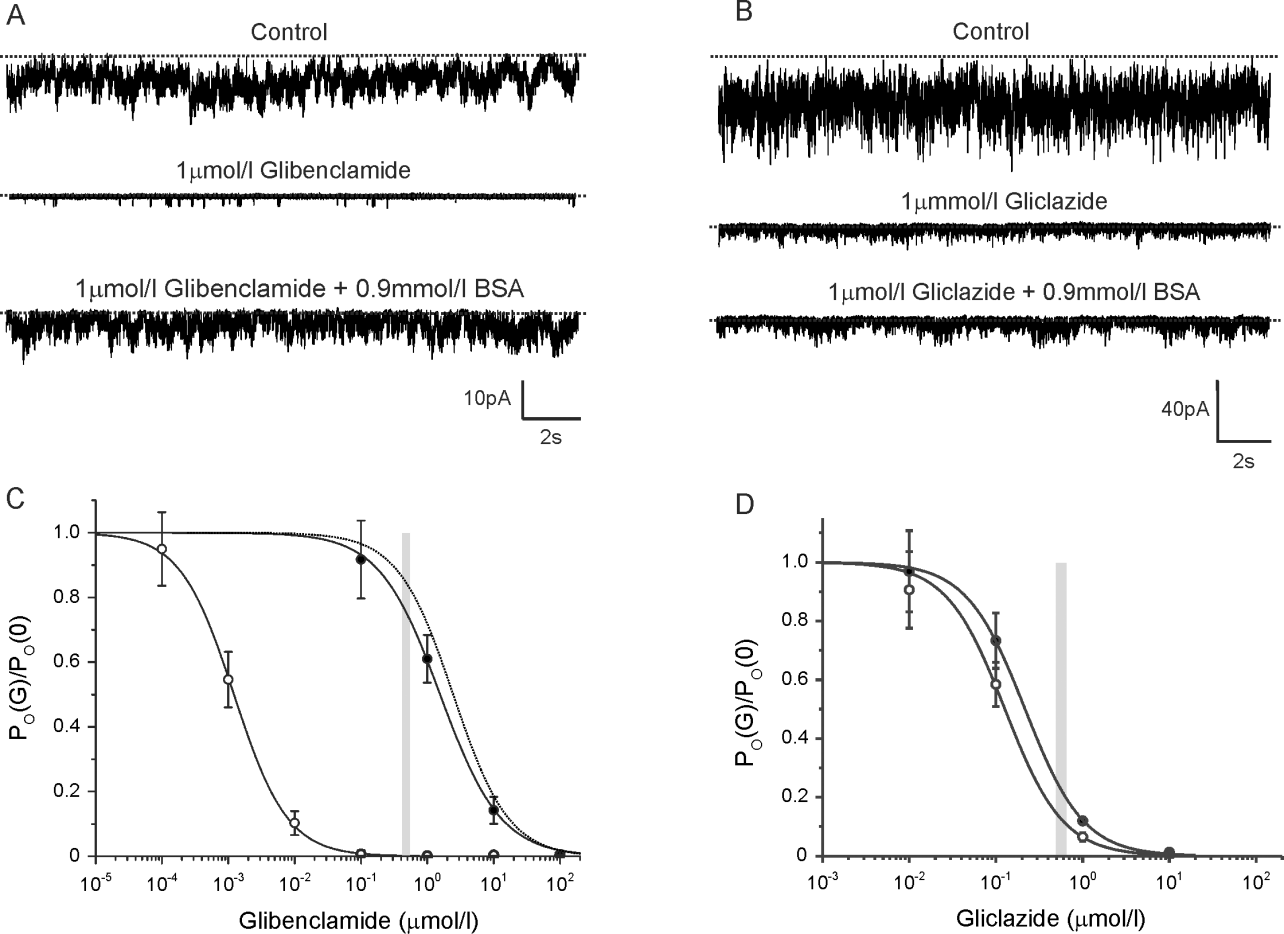
Effect of BSA on glibenclamide and gliclazide block of recombinant β-cell K_ATP_ channels. (A,B) Representative Kir6.2/SUR1 currents recorded at -60mV from cell-attached patches on *Xenopus* oocytes. Currents were recorded in the presence of 3mmol/l Na-azide (top trace), 3mmol/l Na-azide plus 1μmol/l glibenclamide (A, middle) or 3mmol/l Na-azide plus 1μmol/l gliclazide (B, middle), and 3mmol/l Na-azide, 1μmol/l glibenclamide and 0.9mmol/l BSA (**A**, bottom) or 3mmol/l Na-azide, 1μmol/l gliclazide and 0.9mmol/l BSA (**C**, bottom). The dotted line indicates the zero current level. (C) Concentration-response relationships for glibenclamide inhibition of Kir6.2/SUR1 currents in the absence (○, n = 10) and presence (●, n = 10) of 0.9mmol/l BSA. Open probability (*P*_*O*_) was recorded in the cell-attached configuration and is expressed relative to that in the absence of glibenclamide. The lines are the best fit of Eq 2 to the mean data: (○) *IC*_*50*_ = 1.2nmol/l, *h* = 1.1; (●) *IC*_*50*_ = 1.6μmol/l, *h* = 0.95. *a* was set at 0 in both cases. The dotted line is the estimated glibenclamide block in the presence of human plasma (HP) assuming the drug binds to plasma proteins with a *K*_*d*_ of 0.44μmol/l. The width of the grey bar indicates the mean C_SS_±SEM of the total glibenclamide concentration in the plasma of patients with type 2 diabetes [28]. (D) Concentration-response relationships for gliclazide inhibition of Kir6.2/SUR1 currents in the absence (○, n = 10) and presence (●, n = 10) of 0.9mmol/l BSA. Open probability (*P*_*O*_) was recorded in the cell-attached configuration and is expressed relative to that in the absence of gliclazide. The lines are the best fit of Eq 2 to the mean data: *IC*_*50*_ = 128nmol/l, *h* = 1.3 (○); *IC*_*50*_ = 217nmol/l, *h* = 1.3 (●). *a* was set at 0. The *IC*_*50*_ obtained in the absence of BSA (128nmol/l) is similar to that previously reported for whole-cell Kir6.2/SUR1 currents in oocytes (108nmol/l; [27]). The width of the grey bar indicates the mean C_SS_±SEM of the total gliclazide concentration in the plasma estimated from a daily dose of 80mg and an AUC of 44μg.h/ml [26].

In control solution, the mean *P*_*O*_ was 0.32±0.08 (n = 15). Fig 2A shows typical currents recorded from cell-attached patches in control solution (top trace), in the presence of 1μmol/l glibenclamide (middle) and in the presence of 1μmol/l glibenclamide plus 0.9mmol/l BSA (bottom). Fig 2C shows the mean glibenclamide concentration-inhibition curve. In the absence of BSA, the *IC*_*50*_ was 1.2nmol/l, which is close to that reported for inhibition of whole-cell K_ATP_ currents in pancreatic β-cells [1,18]. BSA substantially reduced glibenclamide block, the *IC*_*50*_ increasing to 1.6μmol/l. This suggests that BSA reduced the free glibenclamide concentration more than a thousand-fold, giving a predicted *K*_*d*_ of 0.67μmol/l.

For comparison, we also determined the concentration-response relationship for gliclazide block of β-cell K_ATP_ channels expressed in *Xenopus* oocytes, by measuring *P*_*O*_ in cell-attached patches (Fig 2B–2D). In the absence of BSA, the *IC*_*50*_ was 127nmol/l, which is similar to that obtained previously for whole-cell currents in oocytes (108nmol/l; [27], and in pancreatic β-cells (190nmol/l, Fig 1). The presence of BSA produced a small (non-significant) increase in *IC*_*50*_ to 217nmol/l, which is consistent with the data obtained in β-cells (Fig 1).

### Insulin secretion

**Fig 3:**
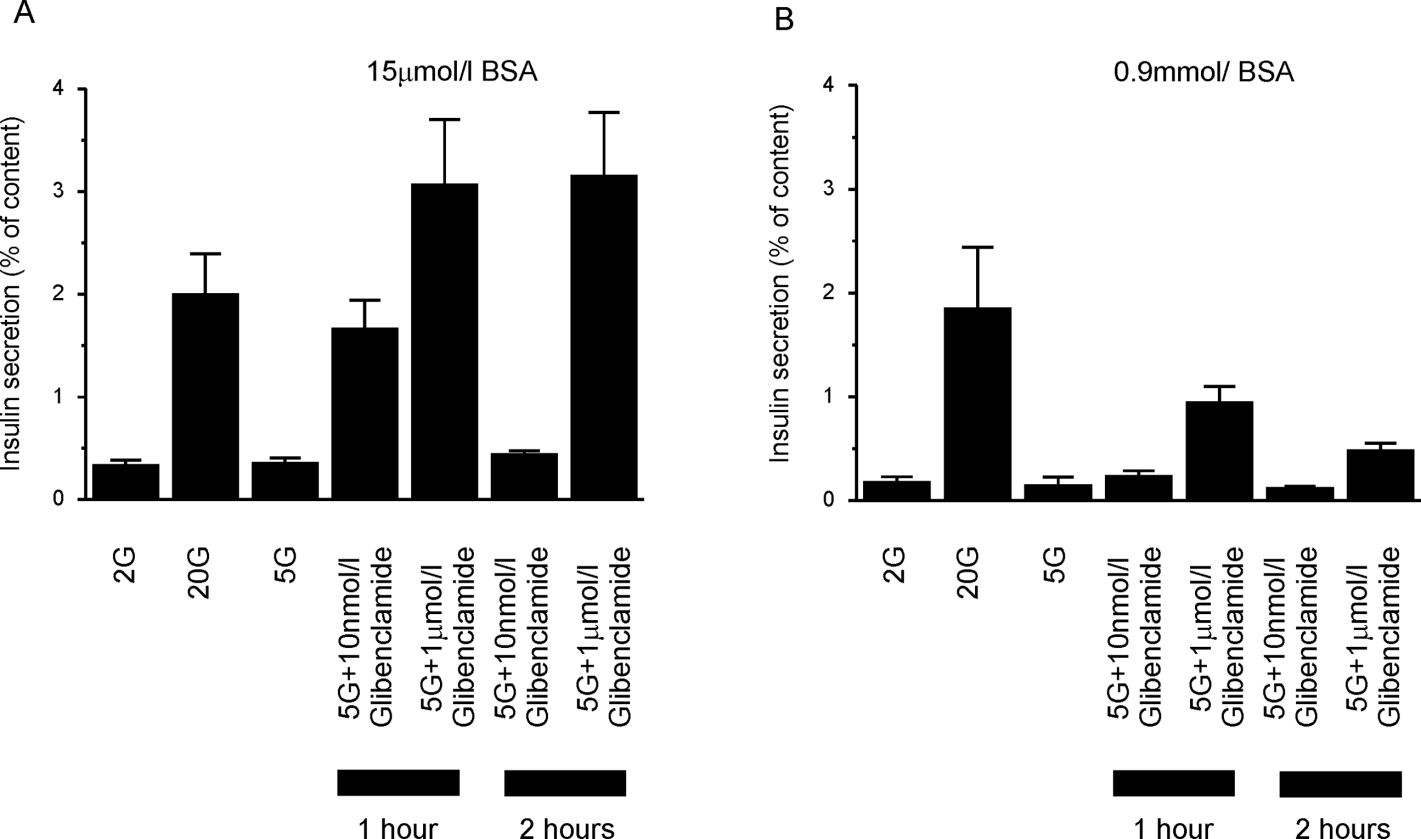
Effect of BSA on glibenclamide stimulated insulin release. Insulin secretion was evaluated in islets from 8–10 week-old male mice in response to 60–120 minute stimulation with various glucose and glibenclamide concentrations, as indicated, in the presence of (A) 15μmol/l BSA or (B) 0.9mmol/l BSA (n = 3 animals, 3 technical replicates per condition). Insulin secretion is expressed as a percentage of the insulin content. Insulin secretion in the presence of glucose and glibenclamide was significantly affected by the BSA concentration (*F(5*,*12)* = 10.77; *p* = 0.004). For both incubation times and for all drug concentrations tested, insulin secretion was significantly lower in the presence of 0.9mmol/l BSA than 15μM BSA (*p*<0.05).

Glucose-stimulated insulin secretion from isolated islets was not significantly affected by 0.9mmol/l BSA (Fig 3), although there was a trend towards reduced basal secretion at 2 and 5mmol/l glucose. We examined the effect of BSA on glibenclamide-stimulated insulin secretion at 5mmol/l glucose, as this is similar to the fasting glucose concentrations in humans. Fig 3 shows that 0.9mmol/l BSA produced a substantial reduction in insulin secretion in response to 1μmol/l glibenclamide, which was ~3-fold lower than in the presence of 15μmol/l BSA. This difference persisted even when the incubation period was extended to 2 hours. Thus BSA markedly reduces the effect of glibenclamide on insulin release as well as on K_ATP_ channel activity.

Previous studies have shown that albumin acts as a Zn^2+^ chelator and causes dissociation of the Zn-insulin complex which enables insulin to be measured correctly (Zn-insulin is not detected by the immunoassay) [29,30]. In our experiments increasing albumen from 15μM to 900μM did not affect insulin release measured in response to 20mM glucose in the absence of glibenclamide (Fig 3). This suggests 15μM albumen must be sufficient to dissociate all Zn^2+^ from insulin. The maximal glibenclamide concentration we used for our experiments was 1μM. This is much less than the albumen concentration (15μM), suggesting glibenclamide should not compromise Zn^2+^ chelation (or insulin measurements).

If we assume that 1μmol/l glibenclamide is a maximally effective stimulus for K_ATP_ channel closure at low BSA levels, as seems reasonable from the concentration-response curves in both oocytes (Fig 2C) and pancreatic β-cells [18], then 10nmol/l glibenclamide produces ~50% stimulation of insulin release (Fig 3A). This is in reasonable agreement with the *K*_*d*_ for glibenclamide binding to SUR1 seen by some authors (4nmol/l, [31]) although others have reported *K*_*d*_ values ~10-fold lower [32]. The *IC*_*50*_ for K_ATP_ channel inhibition is also reasonably close, varying from 1-5nmol/l [1, 2, 18], Fig 3A). By contrast, in the presence of 0.9mmol/l BSA, insulin secretion is only ~30% of maximal (Fig 3B). A corresponding 30% reduction in K_ATP_ current is produced by ~700nmol/l glibenclamide.

### Estimated effect of human plasma on sulphonylurea block of the K_ATP_ channel

We next determined the free concentrations of glibenclamide and gliclazide in human plasma. The percentage of free drug was 14.7±1.5% (n = 3) for gliclazide and 0.05±0.02% (n = 3) for glibenclamide. These values predict dissociation constants (*K*_*d*_) for drug binding to human plasma proteins of 0.44μmol/l for glibenclamide and 155μmol/l for gliclazide (calculated using Eq 3). Thus, the *K*_*d*_ for glibenclamide binding to BSA estimated from electrophysiology (0.67μmol/l) was similar to that for human plasma; in contrast, the K_d_ for gliclazide binding to BSA estimated from the statistically insignificant shift in the *IC*_*50*_ was very different: 1.3mmol/l compared with 155μmol/l for human plasma.

Using the measured *K*_*d*_ values for human plasma, we estimated the relationship between gliclazide (Fig 1C, dotted line) or glibenclamide (Fig 2C, dotted line) and K_ATP_ channel inhibition in presence of human plasma (assuming an albumin concentration in plasma of 60g/l). The predicted glibenclamide sensitivity in the presence of human plasma was similar to that obtained experimentally in the presence of BSA. In contrast, the predicted gliclazide block in the presence of human plasma was significantly lower than that measured in the presence of BSA, presumably because of the difference in the affinity of BSA and human plasma for gliclazide.

Steady-state total plasma concentrations of sulphonylurea drugs (C_SS_) have been used previously as a proxy for the drug concentration encountered at the site of action [33]. The grey bars indicate the estimated circulating C_SS_ of the drug in patients with type 2 diabetes treated with gliclazide ([26]; Fig 1C, Fig 2D) or glibenclamide ([28]; Fig 2C). This reveals that in the presence of BSA K_ATP_ channels are almost fully blocked (>90%) by therapeutic (C_SS_) levels of gliclazide, whereas they are only blocked by ~75% at C_SS_ levels of glibenclamide.

## Discussion

Our binding data confirm that sulphonylureas bind very tightly to plasma proteins, such as BSA and human serum albumin (HSA). We found that 99.9% of glibenclamide is bound, in agreement with earlier studies of drug binding to fatty acid-free HSA [34,35]. Gliclazide also binds to plasma proteins, albeit less tightly than glibenclamide—at the C_SS_, 85% of gliclazide is bound, when binding to human plasma is measured. Interestingly, considerable variability in the affinity of gliclazide binding to HSA (or BSA) is reported in the literature, with the percentage of free drug ranging from less than 1% [9, 36] to 70% [37]. The most likely explanation for this variability is that gliclazide binding to albumin is strongly affected by the composition of solutions used to measure binding [38]. Our data also suggest that whereas glibenclamide binding to human plasma can be largely attributed to albumin, other plasma proteins may contribute to gliclazide binding.

**Fig 4:**
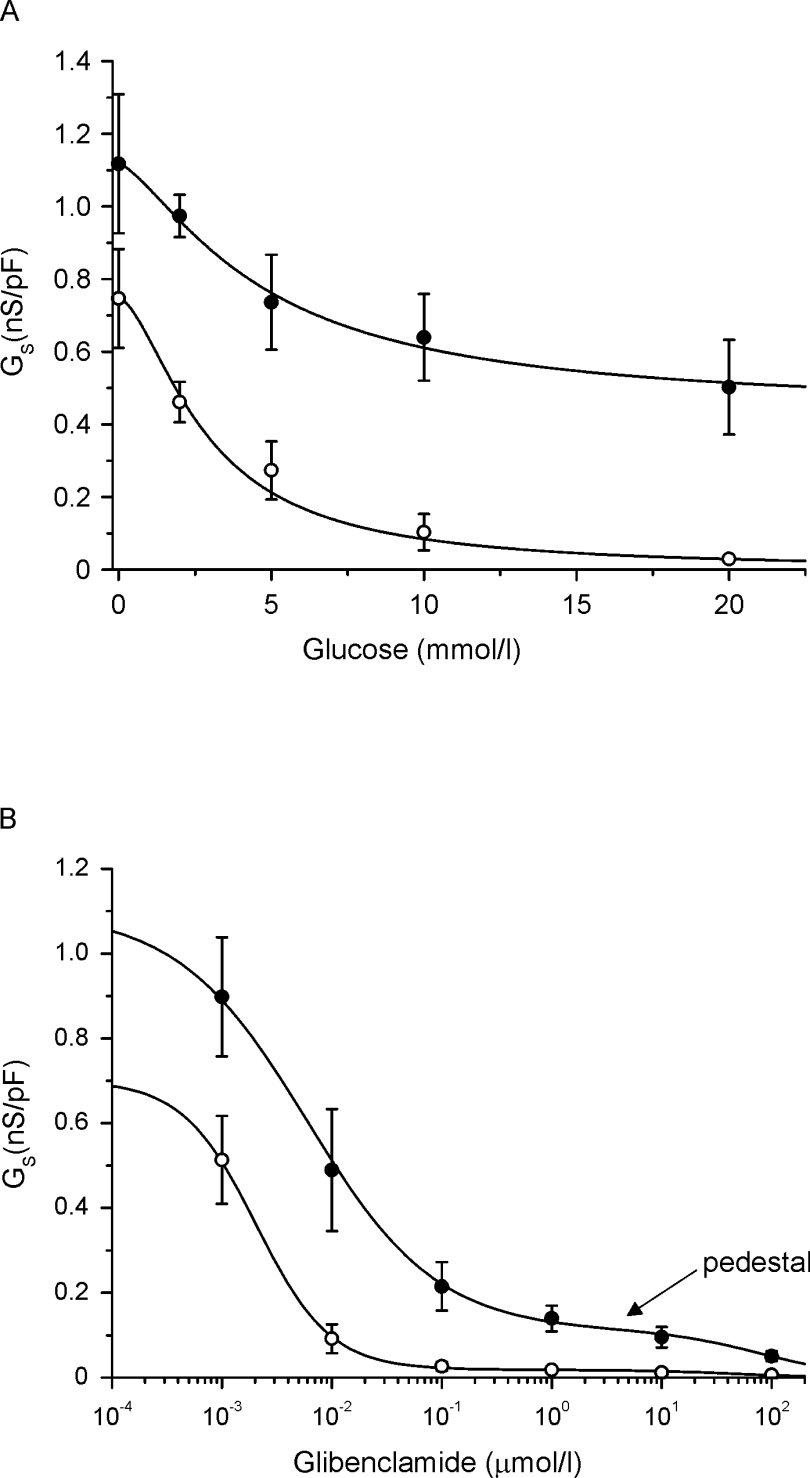
Glucose and glibenclamide block of the K_ATP_ channel. (A) Concentration-response relationships for glucose inhibition of K_ATP_ conductance in pancreatic β-cells from wild-type (○, n = 5) and βV59M (●, n = 8) mice, measured using the perforated patch configuration. Data are the same as those in [16] but are replotted as nS/pF. (B) Concentration-response relationships for glibenclamide inhibition of K_ATP_ conductance in pancreatic β-cells from wild-type (○, n = 6) and βV59M (●, n = 6) mice, measured using the perforated patch configuration. Data are the same as those in [18] but are replotted as nS/pF. Note the ‘pedestal’ (arrowed) in the dose-response curve for glibenclamide inhibition. This results because glibenclamide binds to SUR1 with high affinity and acts a partial antagonist of the K_ATP_ channel, with a maximal block of ~60–80% [14]. It also produces a low affinity block at Kir6.2. However, like other sulphonylureas [39], glibenclamide also displaces MgADP from NBD2 of SUR1, which prevents MgADP activation and thereby reveals the full extent of ATP block at Kir6.2. Thus, in the presence of intracellular nucleotides, inhibition is the sum of the high-affinity glibenclamide block at SUR1, ATP block at Kir6.2, and a low-affinity block by glibenclamide at Kir6.2.

How do these values translate into inhibition of the K_ATP_ channel? Sulphonylureas produce a concentration-dependent decrease in the K_ATP_ current, which depolarises the β-cell membrane and ultimately (once the threshold is exceeded) stimulates electrical activity. Glucose acts in a similar manner. In both cases, it is the actual magnitude of the current (rather than the percentage block) that determines its impact on the membrane potential, and thus insulin secretion. Fig 4 shows concentration-response curves for glucose (A) and glibenclamide (B) inhibition of the K_ATP_ current, measured using the perforated patch configuration to maintain metabolism and retain other regulators of channel activity. The data are expressed as a fraction of the cell capacitance (nS/pF) to compensate for differences in cell size, and shown for both control β-cells and β-cells from mice carrying the Kir6.2-V59M mutation found in patients with neonatal diabetes (βV59M mice). Data have been recalculated from [16] and [18].

Measurements of glibenclamide levels in βV59M mice treated with sulphonylureas range from ~230nmol/l (0.25mg/21 day pellet) to 1μmol/l (2.5mg/21 day pellet; [24]). This corresponds to a free glibenclamide concentration of 0.11nmol/l and 0.5nmol/l, respectively (assuming a *K*_*d*_ of 0.44μmol/l), or a whole-cell K_ATP_ conductance of 1.05nS/pF or 0.96nS/pF (Fig 4B). In both cases, this is far greater than the threshold level for electrical activity, which occurs when glucose exceeds ~6mmol/l, corresponding to ~0.2nS/pF (Fig 4A). Even if we consider that glucose also produces some reduction in the K_ATP_ current of βV59M β-cells, the decrease is not sufficient to initiate electrical activity. This is inconsistent with the fact that glibenclamide produces excellent control of blood glucose in βV59M mice [40].

A similar problem arises when we consider the glibenclamide concentration in human patients. The free glibenclamide level is around 0.2nmol/l in people with type 2 diabetes (calculated from a total concentration of 0.48μmol/l [28], and can be as much as 0.4nmol/l in patients with neonatal diabetes (calculated from a total concentration of 435ng/ml or 880nmol/l; [41]). This corresponds to a block of the K_ATP_ current of ~10% (type 2 diabetes) and 16% (neonatal diabetes). Insulin secretion and electrical activity are stimulated at lower glucose levels in human β-cells (3mmol/l; [42,43], probably because they possess very little GLUT2 [44]. Even if we use the mouse data to estimate the K_ATP_ current at the free drug concentration found in human plasma (0.67pS/pF and 0.62pS/pF, respectively for type 2 diabetes and neonatal diabetes) this is still too large to quantitatively explain the therapeutic effect of the drug. The situation is even worse when we consider that a child born to a diabetic mother treated with glibenclamide had hypoglycaemia, despite an estimated free plasma glibenclamide level of 10pM (20nmol/l total; [41]). This is expected to reduce the wild-type current by less than 1%.

This suggests the effective glibenclamide concentration must be far greater at its site of action than that measured in plasma. A free concentration of at least 100nmol/l is needed in βV59M mice and 40nmol/l in wild-type mice ([18]; Fig 4B). Two possible explanations for this discrepancy can be excluded. First, recent studies indicated that sulphonylurea binding to albumin is altered by glycation of the protein [45]. However, the effect of glycation on glibenclamide binding to HSA appears to be variable, as both an increase [33] and a decrease [46] have been reported. In addition, no obvious effect of albumin glycation on glibenclamide binding was seen with plasma samples from patients with type 2 diabetes [47]. Secondly, glibenclamide is known to accumulate progressively inside β-cells [48]. This is expected to increase its effective concentration at its binding site, which lies within the intracellular domains of SUR1 [3]. However, this is unlikely to account for the effects we observed as glibenclamide uptake is nearly saturated after 30mins [49], whereas glibenclamide-stimulated insulin secretion was still substantially less after 1 hour in the presence of 0.9mmol/l BSA than 15μmol/l BSA, and increasing the incubation time to 2hrs did not further enhance insulin release (this study). There is thus a discrepancy between the *in vitro* and *in vivo* data. A similar discrepancy is seen for other drugs that bind very tightly to plasma proteins, and is hypothesised to result from multiple variables that affect the dynamic free concentration of the drug *in vivo* [50]. Whatever the explanation for the difference, the data mean it is not possible to deduce the magnitude of the K_ATP_ current *in vivo* from the glibenclamide concentration needed to produce euglycaemia.

The free glibenclamide concentration in βV59M mice must be at least 100nmol/l if it is to stimulate insulin release and control the animal’s diabetes. If we assume this is the case, it is obvious why a 10-fold higher dose does not cause hypoglycaemia. The shape of the dose-response curve is such that only a small reduction in current is produced (to ~0.15nS/pF). Presumably the same explanation accounts for the fact that neonatal diabetes patients with the same mutation rarely suffer from hypoglycaemia, even when taking high doses of glibenclamide. The data also explain why hypoglycaemia is more common in patients with type 2 diabetes, who have wild-type K_ATP_ channels, or in neonatal diabetes with less severe K_ATP_ channel mutations that do not give rise a pedestal. The threshold for electrical activity (0.2nS/pF) lies on a steep part of the glibenclamide concentration-response curve, and a 10-fold increase in the drug concentration will result in almost total block of the channel.

Electrical activity in mouse β-cells is initiated when the glucose concentration exceeds 6mmol/l glucose [51], where the K_ATP_ conductance is approximately 0.2 pS/pF (Fig 4A). A similar current magnitude can be expected at the threshold potential in human β-cells. Assuming a daily dose of 80mg and an AUC of 44μg.h/ml [26], the estimated free gliclazide concentration in the plasma of patients with type 2 diabetes is around 855nmol/l (assuming 85% is bound). This will reduce the wild-type K_ATP_ current in pancreatic β-cells by ~75% [52] to 0.19nS/pF, even in the absence of glucose. This is very close to the threshold for electrical activity (0.2nS/pF). Thus it seems that, even in the presence of plasma protein, gliclazide block is quantitatively sufficient to stimulate insulin secretion and the functional data are in reasonable agreement with the free C_SS_ concentration of drug in the plasma of patients with type 2 diabetes. Interestingly, this calculation does not include any potential inhibition of the K_ATP_ current by glucose. This is consistent with the fact that ATP levels do not increase in response to glucose in βV59M isolated from patients with type 2 diabetes [53], which presumably means glucose produces little reduction in K_ATP_ channel activity.

## Conclusions

Our results indicate that free plasma concentration of gliclazide in humans is sufficient to quantitatively explain the enhanced insulin secretion as primarily due to K_ATP_ channel inhibition. However, a substantially higher concentration of glibenclamide at its site of action is required. Our data also stress the importance of unknown factor(s) that influence the free concentration of gliclazide in human plasma and thus its ability to inhibit K_ATP_ channels.

## Supporting information

S1 Dataset Fractional remaining K_ATP_ current in the presence of various gliclazide concentrations measured in the absence and presence of BSA (Fig 1C). (XLSX)

S2 Dataset Cell-attached open probabilities of K_ATP_ channels in *Xenopus* oocytes pre-treated with 3mM Na-azide and different glibenclamide (Fig 2C) or gliclazide (Fig 2D) concentrations either in the absence or presence of BSA. (XLSX)

S3 Dataset Insulin secretion measured in the presence of 15μmol/l BSA (Fig 3A) and 0.9mmol/l BSA (Fig 3B) at various glucose and glibenclamide concentrations. (XLSX)

S4 Dataset Free plasma gliclazide and glibenclamide concentrations measured for different ultrafiltrates. (XLSX)

S5 Dataset Whole-cell K_ATP_ conductance in pancreatic β-cells isolated from wild-type and βV59M mice at different glucose (Fig 4A) and glibenclamide (Fig 4B) concentrations. (XLSX)
